# Supplementary material for: Neural and computational mechanisms of momentary fatigue and persistence in effort-based choice
Source: Nat Commun. 2021 Jul 28;12:4593. doi: 10.1038/s41467-021-24927-7 (PMC8319292; doi:10.1038/s41467-021-24927-7)
Supplement: Supplementary file 1 — Supplementary Information [file 41467_2021_24927_MOESM1_ESM.pdf]

Neural and computational mechanisms of momentary fatigue and persistence in effort-based choice

Tanja Müller, Miriam C Klein-Flügge, Sanjay Manohar, Masud Husain, Matthew A J Apps

Supplementary Information

Supplementary Methods

Apparatus

Stimulus presentation and response collection were implemented using Matlab (MathWorks, Inc., USA) and Psychophysics Toolbox extensions. Grip force was measured using an MRI compatible, handheld dynamometer (TSD121B-MRI; BIOPAC Systems, Inc., USA) with padded "squash" tape to reduce discomfort. Analysis was performed in Matlab 2017b and SPM12. Imaging data was collected at the Oxford Centre for Functional Magnetic Resonance Imaging of the Brain (FMRIB) / Wellcome Trust Centre for Integrative Neuroimaging (WIN) using a 3T Siemens Magnetom Prisma MRI scanner with a 32-channel head coil for signal transmission and reception. Choice responses were collected using a 4-button response pad.

Behavioural fatigue rating experiment

40 participants (24 females) with no history of neurological or psychiatric illness and with a mean age of 25.53 years (*SD* = 5.63; range 18-40) were tested on a behavioural task in which participants exerted effort to receive rewards and rated their level of tiredness on each trial. Unlike in the fMRI experiment here they were not able to choose whether to exert the effort

or not, but instead they were required to exert a level of force (or rest) to receive rewards. After exertion and receiving the reward they were required to rate their level of tiredness (Supplementary Figure 5). Written informed consent was obtained from all participants and the research was approved by the South Central – Oxford A Research Ethics Committee (18/SC/0448).

The experiment consisted of three parts: i) a *Calibration* phase to account for individual differences in strength, which was completed before the experiment was explained in full to the participants, ii) a *Training* phase in which participants familiarised themselves with the effort levels used in this task, and iii) the *Main task*. In the Main task, participants were asked on every trial to rest or to exert force for rewards (credits). Effort and reward levels were identical to the ones used in the main task of the fMRI experiment. Participants were instructed to collect as many credits as they could throughout the experiment, with the total number of credits collected throughout the task determining their payment. That is, participants were paid £8 for their time and received a bonus payment of up to £4 which was proportional to the credits they had earnt in the task.

During *Calibration*, each participant's MVC was measured by squeezing a hand-held dynamometer on three consecutive trials with their dominant hand. Participants were required to apply as much force as possible on each trial, and they received strong verbal encouragement while squeezing. During each attempt, a bar presented on the screen provided feedback of the force being generated. In the second and third attempts, a benchmark representing 105% and 110%, respectively, of the previous best attempt was used to encourage participants to improve on their score. The maximum level of force generated throughout the three attempts was used as MVC.

In the *Training* phase participants practiced reaching each of four effort levels (0, 30, 39, and 48% of each participant's MVC). The trial was successful only when the force generated by the participant exceeded the required level for a sum total of at least 3 seconds in a five-second window. Each trial commenced with a cue in the form of a pie chart, with the number of red segments indicating the upcoming effort level. To make sure that participants carefully and successfully completed this training, they were awarded one credit for each successful squeeze, while they received zero credits for a failure. In an additional four trials, participants practiced manipulating the rating scale before they completed four full practice trials consisting of the different effort levels and a rating in order to familiarise themselves with the task.

The *Main task* (Supplementary Figure 5) consisted of 120 trials, each requiring participants to either rest or work for credits. Work trials consisted of one of three different effort levels, represented by two to four filled segments in a pie chart (cue) that corresponded to 30, 39, and 48% of each participant's MVC. Rest trials were indicated by one filled segment in a pie chart. The cue only indicated the effort level and not the reward. Rewards were presented for 1.5 seconds and only shown to the participants after they had worked or rested on that trial. Effort and reward levels were varied independently and presented in a pseudo-random order to ensure that 10 repetitions of each effort/reward combination were distributed evenly across the task, and each participant was presented with the same sequence to ensure that any potential differences in behaviour could be attributed to individual characteristics.

After this cue, participants were required to rest or to exert the respective force on the dynamometer for at least 3 out of 5 seconds in order to receive the credits. For this purpose, participants were presented with a vertical bar that provided them with real-time feedback

on their force. The target effort level was indicated by a yellow line superimposed on the bar. If participants had to rest on that trial, the bar was presented for the same duration but with the yellow line displayed at the bottom of the bar. Following this, participants were shown the credits they had obtained dependent on their success or failure on that trial. They then were asked to indicate how tired they felt on a scale ranging from 0 to 100, with 0 representing "not tired at all" and 100 representing "completely exhausted". Immediately before the first trial, participants were given as much time as they needed to indicate how tired they currently felt (baseline rating). On each subsequent trial, the starting value on the scale was the value the participant had entered on the previous trial, and participants had a maximum of 5 seconds to either confirm or change this value. Participants could change the value on the rating scale in increments of 1 by using the left and right arrow keys on a keyboard. They then confirmed their chosen value by pressing the downward arrow key, and a green frame appeared around the rating scale. To ensure that participants reported their feelings of exhaustion accurately, it was made clear to them that none of their ratings would have an effect on the task they were asked to complete.

Fatigue rating experiment analysis

The main aim of this behavioural experiment was to examine whether fatigue ratings would be susceptible to the same short-term recoverable and long-term unrecoverable factors that were found to influence the choice data in the fMRI experiment. Because "effort" is not a continuous variable in this experiment, with 0% effort not continuous between 30-48% force levels, separate analyses were performed for work and rest trials. To examine fluctuations in fatigue ratings from trials *n*-1 to trials *n* in which participants had worked, linear regression models were fitted to each participant's trial-by-trial changes in ratings, with z-scored effort and reward as well as their interaction as predictors. Analysis at the group level was made by performing two-tailed t-tests of normalised beta values (t-scores) against zero. In a

second analysis, to test the effect of rest on recovery, changes in fatigue ratings from trials *n*-1 to trials *n* in which participants had rested were averaged across the task for each participant and significant deviation from zero at the group level was tested with a two-tailed non-parametric Wilcoxon signed-rank test. Only trials *n* in which participants had successfully obtained the credits were included in the analyses. This resulted in the exclusion of *M* = 4.21% (*SD* = 6.60) trials. Confidence intervals (CIs) for t-tests refer to the mean whereas CIs for the Wilcoxon test are based on the Hodges-Lehmann estimate (median).

Modelling trial by trial fatigue ratings

To test whether the computational model fitted to choices in the fMRI experiment could also explain changes in fatigue ratings induced by effort and rest, we fitted the five computational models that predicted fatigue effects to the ratings data (Supplementary Figure 7 & Supplementary Table 5). In the full model, fatigue (F) on each trial (t) was calculated as the sum of a participant's baseline fatigue rating (F_{start}), recoverable fatigue (RF) and unrecoverable fatigue (UF):

$$F_{(t)} = F_{\text{start}} + RF_{(t)} + UF_{(t)} \tag{1}$$

RF increases dependent on the effort (E, effort level 2, 3, or 4) exerted on a trial (Equation 2) and decreases dependent on the time rested (T_{rest}) on a trial (Equation 3):

$$RF_{(t)} = RF_{(t-1)} + (\alpha * \ E_{(t)}) \tag{2}$$

$$RF_{(t)} = RF_{(t-1)} - (\delta * \ T_{\text{rest}(t)}) \tag{3}$$

Individuals differ in the degree to which effort increases their fatigue, as reflected by the subject-specific parameter α, and in how quickly they recover during rest, reflected by the

parameter δ. Unlike RF, UF accumulates depending on the effort exerted across the whole task and is not restored by resting during a trial (Equation 4). The parameter θ represents how quickly different individuals build up fatigue that cannot be easily recovered.

$$UF_{(t)} = UF_{(t-1)} + (\theta * E_{(t)}) \tag{4}$$

Initial RF and UF values were set to 0, with RF and UF subsequently updated on each trial according to the respective model and added to the fatigue level indicated by the respective participant before the start of the main task (baseline rating). Based on theoretical considerations, only parameter values >= 0 and RF estimates >= 0 were allowed.

The fit between the model and the data, as indexed by the sum of squared residuals (RSS) between the participant's ratings and the model's estimates, was optimised using *fminsearch* function in Matlab, i.e. model parameters were changed to minimise the difference between each participant's actual fatigue rating and the fatigue rating predicted by the model for each trial. To maximise the chances of finding global rather than local minima, parameter estimation for the full model and for all alternative models was repeated over a grid of initialisation values, with 6 initialisations (ranging from 0 to 1) per parameter. The optimal set of parameters for each model was used for model comparison.

To verify whether the three parameters used to quantify the effects of fatigue were necessary, alternative models were also fitted to participants' ratings. This included models in which there was an effect of UF only (i.e. θ being fitted) or an effect of RF only (i.e. α and δ being fitted). In addition, two further mathematically plausible, but theoretically unlikely, models were included which used only one parameter to scale the effect of effort and rest on recoverable fatigue (i.e. only α being fitted across both work and rest trials). In one of these models, fatigue was only comprised by this one parameter RF, while in a second

model, fatigue comprised UF plus the one parameter RF. These two models had higher AIC values, and thus worse fits, than versions of the RF model including two separate parameters and are thus not shown in figures. No null model was included as fatigue ratings significantly changed across the experiment (Supplementary Figure 6).

In order to investigate the models' relative ability to predict the behavioural data, model fits were compared using Akaike Information Criterion (AIC) with lower values indicating better fit. AIC was calculated according to the following formula, with n representing the number of observations, i.e. the number of trials, and d representing the number of parameters:

$$\mathrm{AIC} = n * \ln\left(\frac{\mathrm{RSS}}{n}\right) + 2 * d \tag{5}$$

Supplementary Results

Behavioural experiment trial-by-trial fatigue ratings results

We first performed a linear regression on changes in fatigue ratings from trial *n*-1 to trial *n* in which participants had worked, including effort (effort level 2, 3, or 4), reward and their interaction as predictors (with a t-test across participants). The effort just exerted significantly predicted increases in fatigue from one trial to the next, while the reward received did not have a significant effect and there was no significant effort × reward interaction (effort: $t(39) = 11.233$, $p < .001$, Cohen's $d = 1.776$, 95% CI = [2.922, 4.205]); reward: $t(39) = 1.458$, $p = .153$, Cohen's $d = 0.231$, 95% CI = [-0.114, 0.701]; effort × reward: $t(39) = -1.823$, $p = .076$, Cohen's $d = -0.288$, 95% CI = [-0.630, 0.033]; Supplementary Figure 6). In addition, we tested whether participants' change in fatigue ratings across the task from trials *n*-1 to trials *n* in which participants had rested significantly deviated from zero. We found that rests significantly reduced feelings of fatigue across the task ($Z = -4.773$, $p < .001$, 95% CI = [-2.550, -0.817]; Supplementary Figure 6). These results overall suggest

dynamic changes in fatigue resulting from effort, with partial recovery during short periods of rest. Moreover, in line with the hypothesised effects of effort and rest on levels of fatigue, we found that the full model, which predicted effects of fatigue in the choice data collected in the fMRI experiment, was a better fit to trial-by-trial fatigue ratings than the alternative models (Supplementary Figure 7 & Supplementary Table 5). These results support the notion that fatigue has two components, one recoverable and one unrecoverable, that build up during effortful tasks.

fMRI experiment fatigue pre-post task ratings results

These results support the hypothesis that the value ascribed to exerting effort for reward fluctuates over time as a function of 'fatigue' as estimated by the model. However, there are other factors that may correlate with the effects of fatigue within this model, such as boredom or the accumulation of reward. To further demonstrate that this model was capturing sensations of fatigue, we correlated the model parameters for each participant with the change in their subjective ratings of fatigue before and after the main task of the fMRI study using Spearman's rank correlation coefficient[1]. We found a significant correlation between the UF parameter (θ) and the change in rating ($r_s = .361$, two-tailed $p = .033$, 95% CI = [0.032, 0.620]). Participants showing a greater increase in ratings of fatigue had a higher parameter weight, suggesting a greater reduction in the willingness to exert effort for reward due to UF. No significant correlations were identified between the parameter weights defining RF and the change in ratings, although such a result is to be expected as RF putatively only has short-term effects but ratings were taken more than one hour apart.

Could these results be due to participants becoming more random in the main task due to boredom or other confounding factors? We show that effort and reward still have very

[1] The CI reported here follows the z-approximation of Hollander, Wolfe & Chicken (2013).

strong significant effects when examining only the last quarter of trials (effort: $Z = -5.232$; $p < .001$, 95% CI = [-1.916, -1.420]; reward: $Z = 4.305$; $p < .001$, 95% CI = [0.714, 1.548]). As such participants were basing their behaviour strongly on the effort levels towards the end, which is not consistent with more random behaviour. In addition, participants were still choosing to work on almost 100% of trials for the highest reward and lowest effort in the last 27 trials of the main task (see Supplementary Figure 2), which is also inconsistent with fatigue causing more stochastic behaviour.

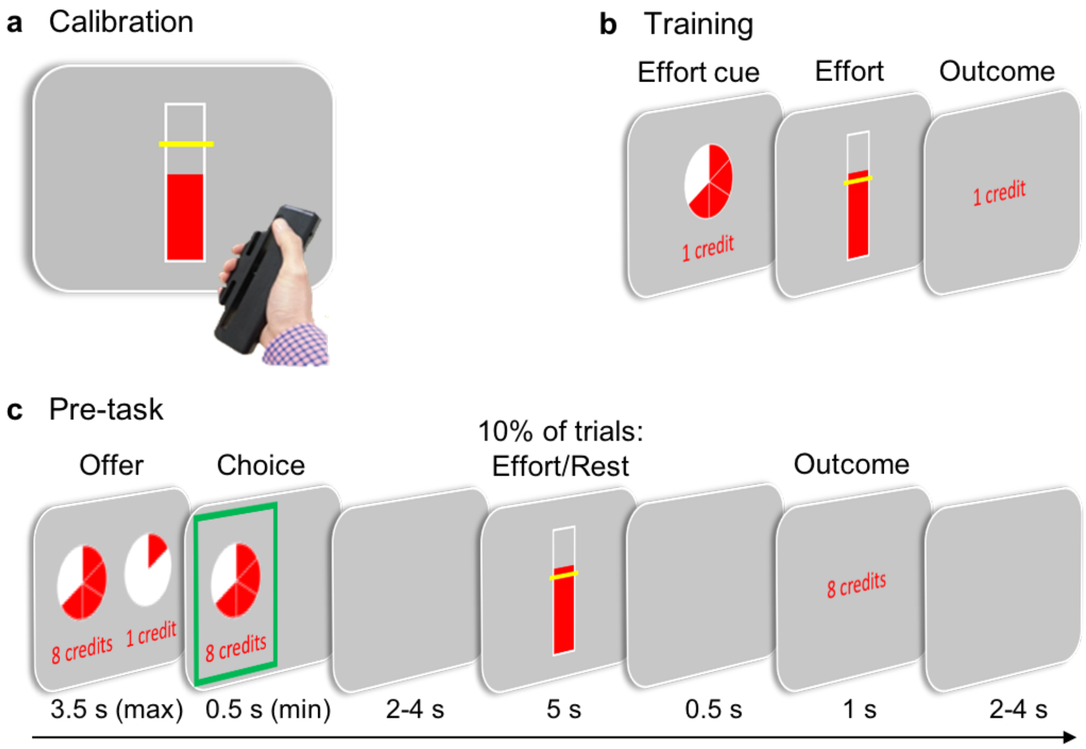

Supplementary Figure 1. Three phases of the task prior to scanning. **a** Participants were required to squeeze a dynamometer as hard as they could in order to calibrate their effort levels. Participants squeezed as hard as they could on a first trial. They were then instructed to get the red fill over the yellow in two more trials, where the yellow line was set at 105% and 110% of the highest contraction value up to that point. The resulting maximum voluntary contraction (MVC) was used to set the effort levels for the rest of the experiment. **b** Participants performed 18 trials of training (3 at each effort level) where they were required to exert one of the six levels of effort (0, 30, 39, 48, 57, 66% MVC) for a sum total of 3s out of 5s in order to receive a credit; if they failed 0 credits were received. Participants were instructed to think about how much force they had to apply at each level and learn to associate each force level with the corresponding pie chat, for which the number of elements indicated the level of force required. **c** In the pre-scanning task participants made choices between a work offer that varied in reward and effort, and a rest offer which never required any force to receive 1 credit. Unlike in the main task, only 10% of randomly selected trials resulted in subsequent requirement to work if chosen. On the remaining trials the screen remained blank after the choice period for an average of 12.5s.

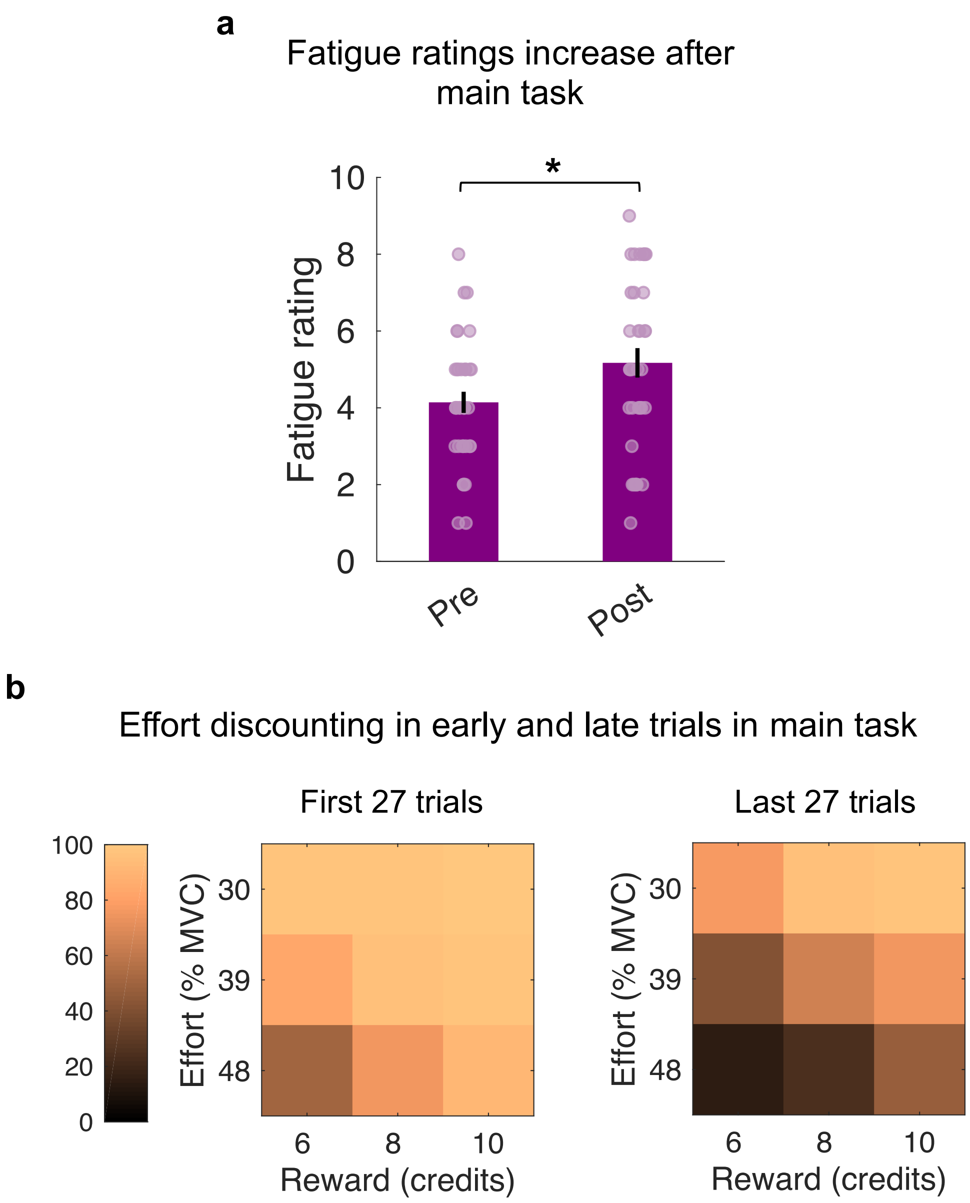

Supplementary Figure 2. **a** Fatigue ratings taken before and after the main task of the fMRI study. Each dot represents one subject and error bars reflect SEM. Fatigue was higher after the main task than before, as indicated by a repeated measures t-test (two-tailed p = .0001; n = 35). **b** Proportions (means) of choices to 'work', illustrating a shift in choices away from higher effort lower reward options in last 27 trials compared to first 27 trials. Shift does not occur for lowest effort highest reward offer, consistent with a shift in valuation not more random behaviour.

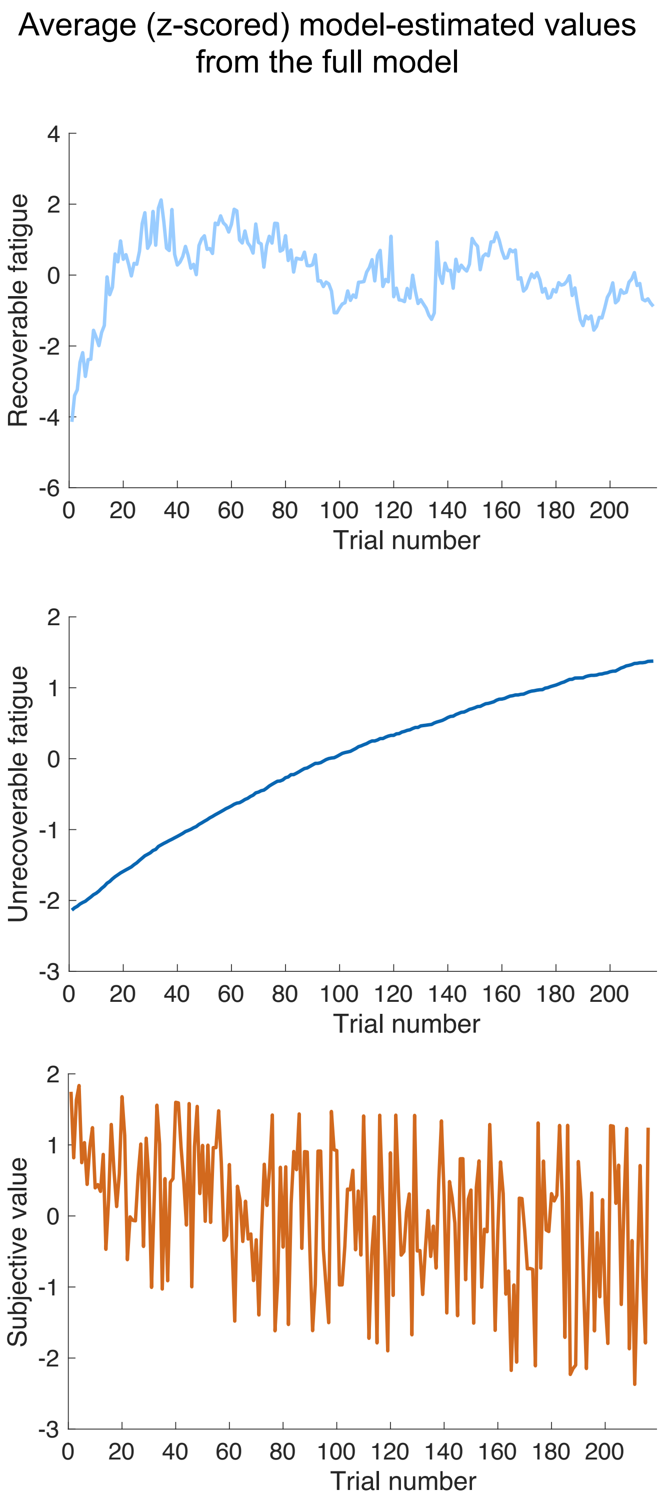

Supplementary Figure 3. Values for recoverable fatigue, unrecoverable fatigue and fatigue-weighted subjective value from full model once fitted separately to all participants' data and then averaged and z-scored.

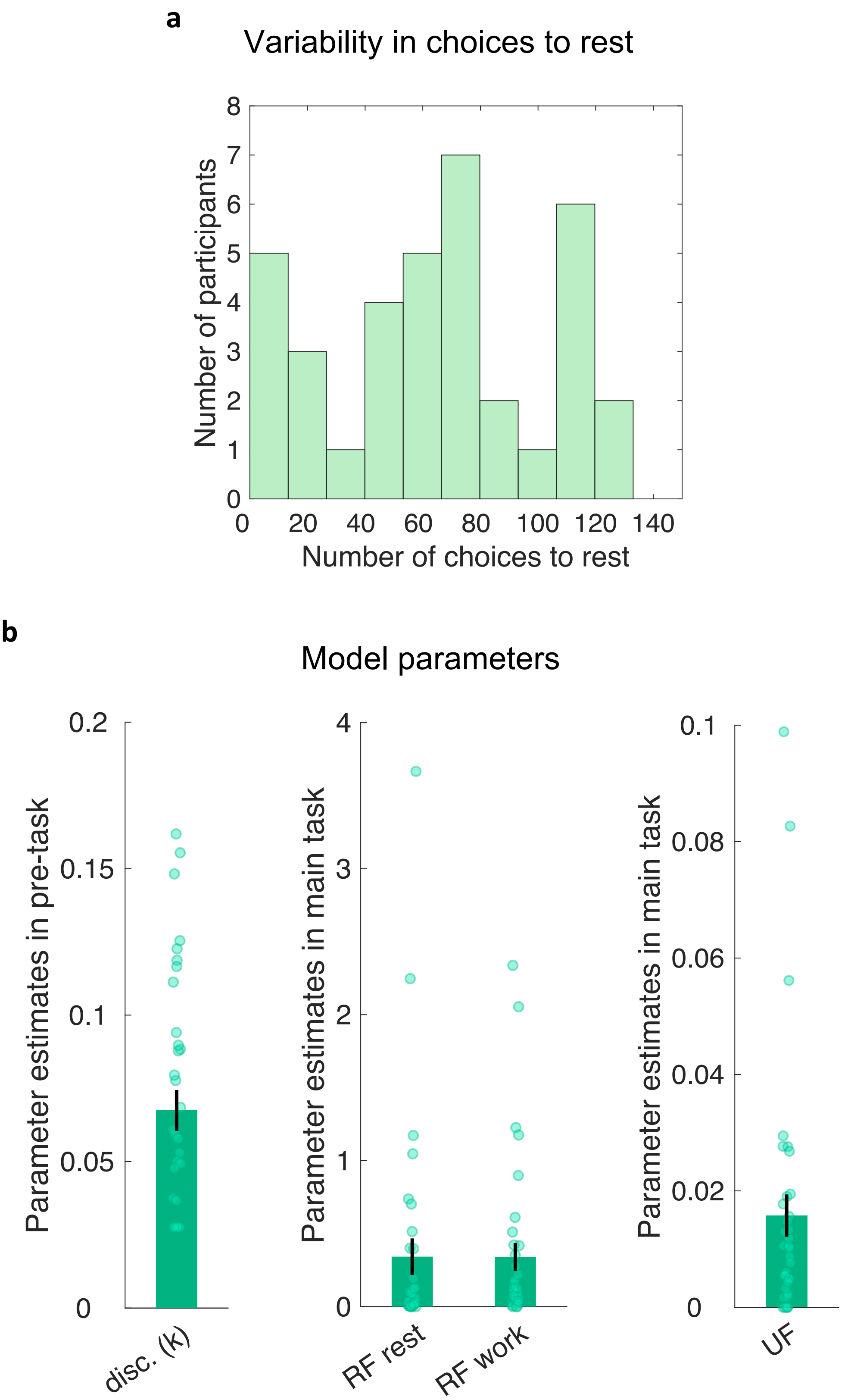

Supplementary Figure 4. Supplementary results from fMRI study behaviour. **a** Histogram of proportion of trials on which participants made choices to 'rest' out of 216 trials in the main task. There is considerable variability in choices both within and between many participants. **b** Model parameters for each participant (green dot) and average across participants for the discounting parameter fitted to the pre-task (left; *n* = 36), the two recoverable fatigue parameters (RF; *n* = 35) and the unrecoverable fatigue parameter (UF; *n* = 36). One participant's RF parameters were not included for display purposes due to high values. Error bars represent SEM.

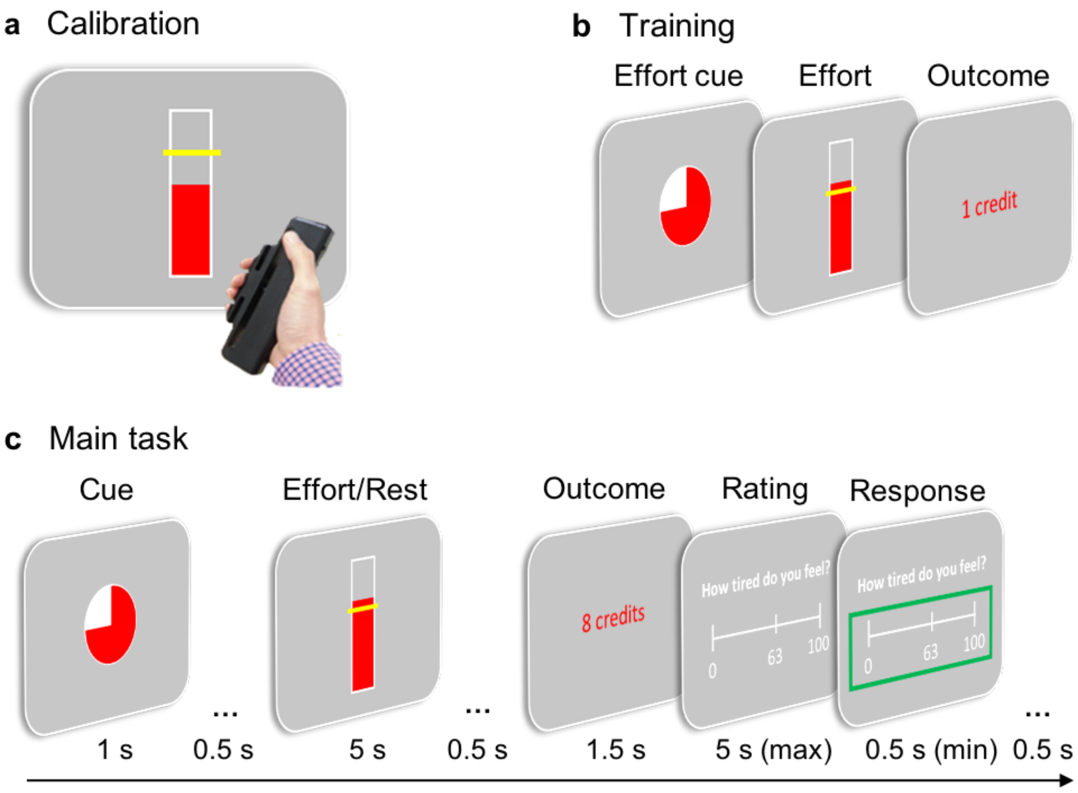

Supplementary Figure 5. Behavioural study trial structure. Participants were required to exert force for rewards, with effort levels calibrated to their maximum voluntary contraction (MVC) (**a**). After training at each of four effort levels (0,30,39,48% MVC) (**b**), they performed 120 forced execution trails (**c**). On each trial they were instructed an effort that would be required (indicated by a pie chart), and then they required to exert that level of force for a total of 3s out of 5s to obtain credits. Participants were then told the amount of credits received – 6, 8 or 10 credits if successful or 0 credits if failing to exert the required force. Following this they rated their level of tiredness from 0-100 on a continuous scale.

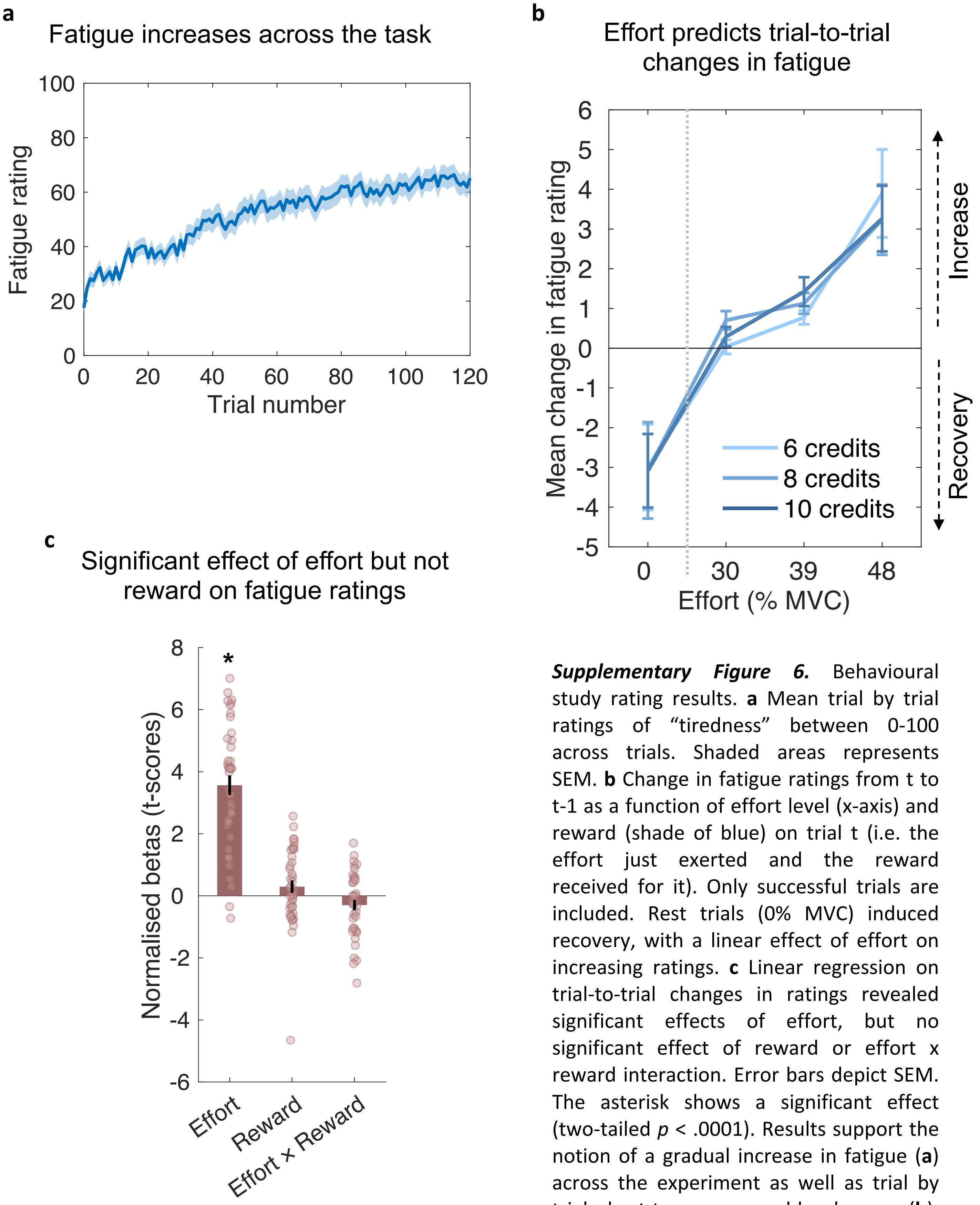

Supplementary Figure 6. Behavioural study rating results. **a** Mean trial by trial ratings of "tiredness" between 0-100 across trials. Shaded areas represents SEM. **b** Change in fatigue ratings from t to t-1 as a function of effort level (x-axis) and reward (shade of blue) on trial t (i.e. the effort just exerted and the reward received for it). Only successful trials are included. Rest trials (0% MVC) induced recovery, with a linear effect of effort on increasing ratings. **c** Linear regression on trial-to-trial changes in ratings revealed significant effects of effort, but no significant effect of reward or effort x reward interaction. Error bars depict SEM. The asterisk shows a significant effect (two-tailed $p < .0001$). Results support the notion of a gradual increase in fatigue (**a**) across the experiment as well as trial by trial short-term recoverable changes (**b**). All $n = 40$.

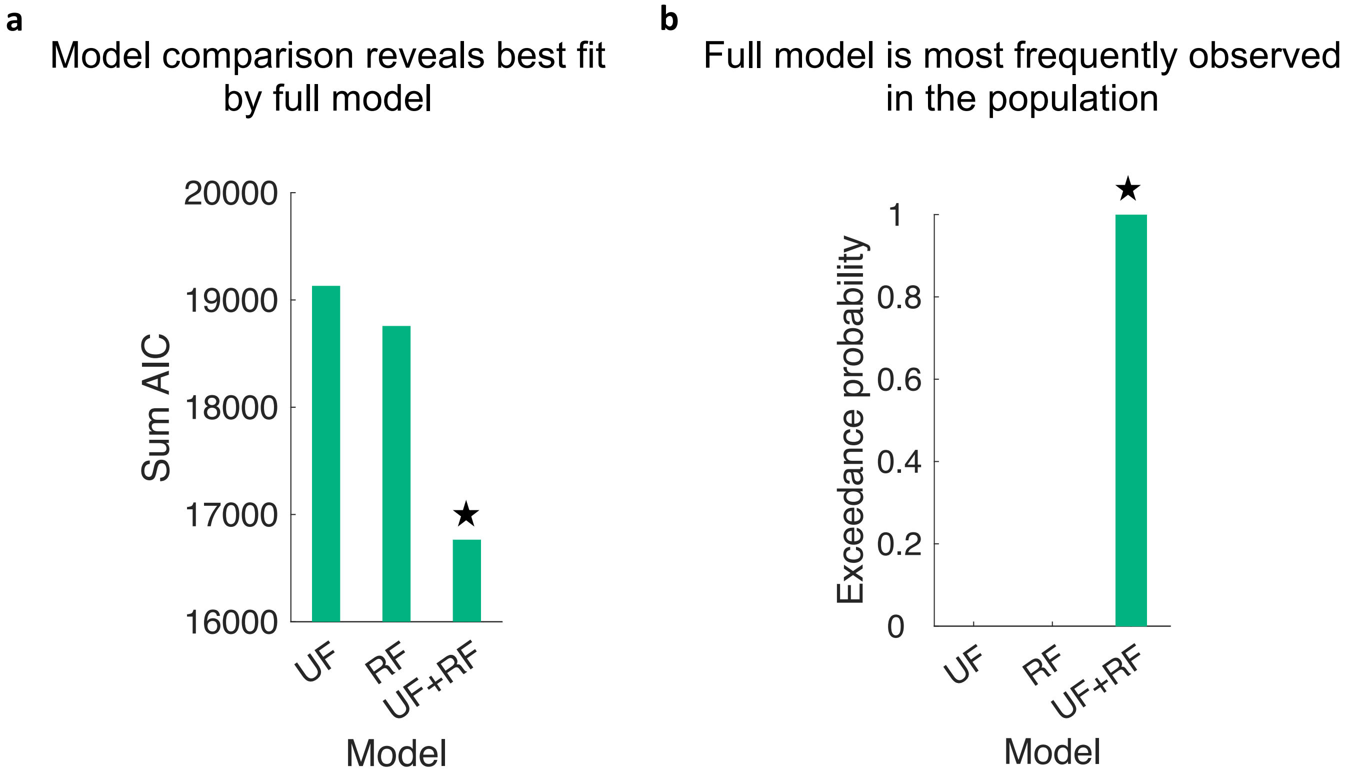

c Models tested

model 1: Unrecoverable fatigue only (UF)

$F = UF$

$UF_{(t)} = UF_{(t-1)} + (\theta * E_{(t)})$

model 2: Recoverable fatigue only (RF)

$F = RF$

$RF_{(t)} = RF_{(t-1)} + (\alpha * E_{(t)}) - (\delta * T_{rest(t)})$

model 3: Full model of fatigue (UF + RF)

$F = UF + RF$

$RF_{(t)} = RF_{(t-1)} + (\alpha * E_{(t)}) - (\delta * T_{rest(t)})$

$UF_{(t)} = UF_{(t-1)} + (\theta * E_{(t)})$

Supplementary Figure 7. Computational modelling results for behavioural rating study. **a** Model comparison results in AIC for the main models predicting changes in fatigue ratings across the experiment. The full model is the best fit to the data when punishing for the number of parameters. **b** Exceedance probabilities for the main models fitted to the ratings data. Y-axis reflects the probability of being the most frequently observed model in the population. Model 3 is the winning "full" model of fatigue containing separate recoverable fatigue (*RF*) and unrecoverable fatigue (*UF*) components. **c** Main models compared. All models predicted changes in fatigue ratings (*F*). The best fitting model (full model) predicted changes in fatigue that were partially recoverable – increasing through effort (*E*) and decreasing through time spent resting (T_{rest}), scaled for each participant by two corresponding free parameters that define a person's short-term fatigability (α, δ) – but also contained a long-term unrecoverable component that increases through exerted efforts (*E*) and never declines in this task, weighted by an idiosyncratic free parameter (θ) defining long-term fatigability. Models 1 and 2 include only the effects of *UF* or *RF*.

Supplementary Table 1

Anatomical locations in which activity significantly covaried with recoverable fatigue, as derived from the computational model, at p < .001, uncorrected for multiple comparisons

Anatomical area	MNI peak	No. of voxels	Z-value	Voxel $p_{uncorr.}$
Right precuneus	24, -46, 26	12535	6.33	< 0.001*
Left inferior temporal gyrus	-42, -10, -34	14	4.02	< 0.001
Right anterior orbital gyrus	27, 41, -19	19	3.97	< 0.001
Right cuneus	6, -94, 20	11	3.84	< 0.001
Left anterior orbital gyrus	-15, 59, -16	6	3.82	< 0.001
Right precentral gyrus	39, -7, 62	42	3.68	< 0.001
Right temporal pole	42, 23, -37	18	3.58	< 0.001
Left temporal pole	-48, 17, -22	15	3.57	< 0.001
Right frontal pole	3, 65, -1	27	3.53	< 0.001
Left temporal pole	-33, 17, -37	23	3.44	< 0.001
Left angular gyrus	-36, -67, 41	14	3.40	< 0.001
Left precuneus	-18, -49, 50	26	3.39	< 0.001

Note. Coordinates are given in Montreal Neurological Institute (MNI) space (x, y, z). A t-contrast was conducted. * indicates significance at a threshold of $p < .05$ with a whole-brain voxel-level family-wise error correction.

Supplementary Table 2

Anatomical locations in which activity significantly covaried with unrecoverable fatigue, as derived from the computational model, at p < .001, uncorrected for multiple comparisons

Anatomical area	MNI peak	No. of voxels	Z-value	Voxel $p_{uncorr.}$
Left precuneus	-3, -67, 32	1852	5.18	< 0.001*
Left calcarine cortex	-12, -97, -1	1584	4.88	< 0.001*
Left middle frontal gyrus	-39, 8, 59	304	4.72	< 0.001*
Left middle temporal gyrus	-69, -37, -7	206	4.64	< 0.001*
Right middle temporal gyrus	69, -25, -10	62	4.43	< 0.001
Right angular gyrus	45, -67, 32	443	4.34	< 0.001
Brain stem	-3, -31, -37	46	3.98	< 0.001
Left middle frontal gyrus	-42, 47, -4	29	3.89	< 0.001
Right inferior temporal gyrus	57, -1, -34	16	3.69	< 0.001
Left RCZa / pre-SMA	-6, 20, 47	15	3.67	< 0.001
Right hippocampus	24, -19, -7	10	3.58	< 0.001
Right middle temporal gyrus	63, -10, -25	8	3.32	< 0.001
Left cerebellum	-6, -58, -16	6	3.27	< 0.001

Note. Coordinates are given in Montreal Neurological Institute (MNI) space (x, y, z). A t-contrast was conducted. * indicates significance at a threshold of $p < .05$ with a whole-brain voxel-level family-wise error correction.

Supplementary Table 3

Anatomical locations in which activity significantly covaried with fatigue-weighted subjective value, as derived from the computational model, at p < .001, uncorrected for multiple comparisons

Anatomical area	MNI peak	No. of voxels	Z-value	Voxel $p_{uncorr.}$
Left cuneus	0, -88, 23	10344	5.75	< 0.001*
Left superior frontal gyrus	-21, 38, 50	390	4.73	< 0.001*
Right anterior orbital gyrus	21, 35, -7	73	3.91	< 0.001
Left temporal pole	-54, 14, -25	13	3.88	< 0.001
Left cerebellum	-27, -52, -55	29	3.77	< 0.001
Right medial frontal cortex	9, 44, -16	12	3.53	< 0.001
Right fusiform gyrus	39, -4, -28	9	3.49	< 0.001
Right superior temporal gyrus	48, -4, -13	9	3.49	< 0.001
Left inferior fontal gyrus	-54, 35, 11	6	3.33	< 0.001
Left occipital fusiform gyrus	-39, -79, -19	7	3.33	< 0.001
Right inferior temporal gyrus	60, -49, -16	8	3.30	< 0.001
Right lingual gyrus	18, -76, -13	9	3.23	< 0.001

Note. Coordinates are given in Montreal Neurological Institute (MNI) space (x, y, z). A t-contrast was conducted. * indicates significance at a threshold of $p < .05$ with a whole-brain voxel-level family-wise error correction.

Supplementary Table 4

Anatomical locations in which activity significantly covaried with individual reaction times at $p < .001$, uncorrected for multiple comparisons

Anatomical area	MNI peak	No. of voxels	Z-value	Voxel $p_{uncorr.}$
Left anterior insula	-33, 17, 5	11158	7.69	< 0.001*
Brain stem	6, -22, -7	367	6.26	< 0.001*
Left inferior temporal gyrus	-45, -58, -13	338	5.32	< 0.001*
Right pallidum	18, 2, -1	82	4.31	< 0.001
Left cerebellum	-36, -52, -34	32	4.28	< 0.001
Right inferior temporal gyrus	48, -61, -13	154	4.23	< 0.001
Brain stem	6, -28, -34	68	4.17	< 0.001
Left medial orbital gyrus	-21, 38, -22	13	3.99	< 0.001
Left medial orbital gyrus	-15, 11, -19	8	3.61	< 0.001
Left thalamus	-12, -22, 14	8	3.55	< 0.001
Right frontal pole	21, 65, -1	6	3.52	< 0.001
Left hippocampus	-24, -25, -4	6	3.44	< 0.001
Right central operculum	48, -19, 17	8	3.32	< 0.001
Right inferior frontal gyrus	51, 47, -10	7	3.29	< 0.001

Note. Coordinates are given in Montreal Neurological Institute (MNI) space (x, y, z). A t-contrast was conducted. * indicates significance at a threshold of $p < .05$ with a whole-brain voxel-level family-wise error correction.

Supplementary Table 5

AIC results (sum) for all models fitted to both choice data and ratings data; RF + UF (recoverable fatigue + unrecoverable fatigue) is the full, hypothesised model of fatigue

Full Model Comparison Results	fMRI choice behaviour AIC	Behavioural rating study AIC
**No change**	61223	N/A
**No Fatigue**	23290	N/A
**UF**	20596	19132
**One parameter RF**	59171	30297
**One parameter RF +UF**	19602	18019
**RF**	17338	18757
**RF + UF**	16832	16765
