# Supplementary material for: Neural and computational mechanisms of momentary fatigue and persistence in effort-based choice
Source: Nat Commun. 2021 Jul 28;12:4593. doi: 10.1038/s41467-021-24927-7 (PMC8319292; doi:10.1038/s41467-021-24927-7)
Supplement: Supplementary file 3 — Reporting Summary [file 41467_2021_24927_MOESM3_ESM.pdf]

nature research

Corresponding author(s): Tanja Müller; Matthew Apps

Last updated by author(s): Jun 30, 2021

Reporting Summary

Nature Research wishes to improve the reproducibility of the work that we publish. This form provides structure for consistency and transparency in reporting. For further information on Nature Research policies, see our Editorial Policies and the Editorial Policy Checklist.

Statistics

For all statistical analyses, confirm that the following items are present in the figure legend, table legend, main text, or Methods section.

n/a	Confirmed	
☐	☒	The exact sample size (n) for each experimental group/condition, given as a discrete number and unit of measurement
☐	☒	A statement on whether measurements were taken from distinct samples or whether the same sample was measured repeatedly
☐	☒	The statistical test(s) used AND whether they are one- or two-sided<br>*Only common tests should be described solely by name; describe more complex techniques in the Methods section.*
☐	☒	A description of all covariates tested
☐	☒	A description of any assumptions or corrections, such as tests of normality and adjustment for multiple comparisons
☐	☒	A full description of the statistical parameters including central tendency (e.g. means) or other basic estimates (e.g. regression coefficient) AND variation (e.g. standard deviation) or associated estimates of uncertainty (e.g. confidence intervals)
☐	☒	For null hypothesis testing, the test statistic (e.g. F, t, r) with confidence intervals, effect sizes, degrees of freedom and P value noted<br>*Give P values as exact values whenever suitable.*
☒	☐	For Bayesian analysis, information on the choice of priors and Markov chain Monte Carlo settings
☒	☐	For hierarchical and complex designs, identification of the appropriate level for tests and full reporting of outcomes
☐	☒	Estimates of effect sizes (e.g. Cohen's d, Pearson's r), indicating how they were calculated

Our web collection on statistics for biologists contains articles on many of the points above.

Software and code

Policy information about availability of computer code

Data collection	Stimulus presentation and response collection were implemented using Matlab 2012a with Psychophysics Toolbox extensions. The code for the experiment is freely available (Digital Object Identifier: DOI 10.17605/OSF.IO/XR84W; link: https://osf.io/xr84w/).
Data analysis	Data analysis was conducted in Matlab 2017b and SPM12.

For manuscripts utilizing custom algorithms or software that are central to the research but not yet described in published literature, software must be made available to editors and reviewers. We strongly encourage code deposition in a community repository (e.g. GitHub). See the Nature Research guidelines for submitting code & software for further information.

Data

Policy information about availability of data

All manuscripts must include a data availability statement. This statement should provide the following information, where applicable:

- Accession codes, unique identifiers, or web links for publicly available datasets
- A list of figures that have associated raw data
- A description of any restrictions on data availability

Source data for Figs. 2b, 2f, 3d, 4c-e, 5a-b and Supplementary Figs. 2a, 3, 4c, 6a-c are provided with this paper as part of the Open Science Framework (OSF; Digital Object Identifier: DOI 10.17605/OSF.IO/XR84W; link: https://osf.io/xr84w/). Unthresholded statistical maps underlying Figs. 4 and 5 are available at the same link. Further fully anonymised behavioural and fMRI data that support the findings of this study are available from the corresponding authors upon reasonable request as they are currently being analysed for future manuscripts.

Field-specific reporting

Please select the one below that is the best fit for your research. If you are not sure, read the appropriate sections before making your selection.

- [ ] Life sciences
- [x] Behavioural & social sciences
- [ ] Ecological, evolutionary & environmental sciences

For a reference copy of the document with all sections, see nature.com/documents/nr-reporting-summary-flat.pdf

Behavioural & social sciences study design

All studies must disclose on these points even when the disclosure is negative.

Study description	This study examined the hidden internal states that lead to changes in the willingness to exert effort over time and their neural correlates. Effort was operationalised as the amount of grip force that needed to be exerted in order to obtain rewards. Data are quantitative experimental data including choice data, self-report fatigue ratings and fMRI measures. In the fMRI study, particpants' primary data was choices between "work" (exerting effort for reward) and "rest" (exerting no effort for a lower reward). In the behavioural study, participants were forced to exert effort (or rest) for reward and then rated their level of fatigue between 0 and 100.
Research sample	A random sample of 39 healthy adults (16 females) took part in the fMRI study. Recruitment criteria included no contraindications that prohibited MRI scanning, no history of neurological or psychiatric disorder, normal or corrected-to-normal vision, right-handedness, and aged 18-40 years. A second random sample in a similar age range of 41 healthy adults (24 females) took part in the behavioural study. The samples included university students and staff as well as members of the public in the vicinity of Oxford. The samples were not representative as this was not possible for the project.
Sampling strategy	Random sampling was used. The sample size was selected based on previous fMRI studies in which similar samples evoked responses in hypothesised regions (Chong et al., 2017, PLoS Biology; Blain et al., 2016, PNAS).
Data collection	Choice responses were collected using a 4-button response pad and grip force was measured using an MRI compatible, handheld dynamometer (TSD121B-MRI; BIOPAC Systems, Inc., USA). Imaging data were recorded using a Siemens Prisma 3T MRI scanner. Self-report fatigue ratings in the fMRI study were recorded using paper and pencil, while self-report fatigue ratings in the follow-up behavioural study were recorded using a keyboard and a computer. During the behavioural study and during the practice and pre-task of the fMRI study, only the participant and the researcher were present in the room. During the main task of the fMRI study, participants performed the task inside an MRI scanner in a room adjacent to the researcher and the radiographer. The researcher was not blind to the study hypotheses, but instructions and procedures were standardised.
Timing	Data collection for the fMRI study took place between May 2017 and February 2018, with no systematic gap between participant collection. Data collection for the behavioural study took place between November 2018 and February 2019, with no systematic gap between participant collection.
Data exclusions	Pre-established exclusion criteria included interruption/premature termination of the experiment and excessive head motion during scanning. n=1 participant terminated the experiment prematurely by pressing the emergency button and was therefore excluded from the fMRI study (see below), and n=2 further participants in the fMRI study were excluded from the analyses due to excessive head motion (more than 6mm of translation). The final sample of 36 participants (16 females) had a mean age of 25.31 years (SD = 4.90; range 18-40). In addition, n=1 participant was excluded from the behavioural study due to recent psychiatric illness. The final sample of 40 participants (24 females) had a mean age of 25.53 years (SD = 5.63; range 18-40).
Non-participation	One participant did not fully complete the fMRI study because of discomfort in the MRI scanner.
Randomization	This was a within-subject design and participants were not allocated to groups.

Reporting for specific materials, systems and methods

We require information from authors about some types of materials, experimental systems and methods used in many studies. Here, indicate whether each material, system or method listed is relevant to your study. If you are not sure if a list item applies to your research, read the appropriate section before selecting a response.

Materials & experimental systems

n/a	Involved in the study	
x		Antibodies
x		Eukaryotic cell lines
x		Palaeontology and archaeology
x		Animals and other organisms
	x	Human research participants
x		Clinical data
x		Dual use research of concern

Methods

n/a	Involved in the study	
x		ChIP-seq
x		Flow cytometry
	x	MRI-based neuroimaging

Human research participants

Policy information about studies involving human research participants

Population characteristics	See above.
Recruitment	Participants were recruited through a departmental database and online bulletin boards. Biases typical for this nature of sampling will be present, but are unlikely to significantly impact on inferences made - most people experience fatigue and find effort aversive and avoid it where possible.
Ethics oversight	The fMRI study was approved by the University of Oxford Medical Sciences Interdivisional Research Ethics Committee and the University of Oxford Central University Research Ethics Committee (MSD-IDREC-C1-2014-037). The behavioural study was approved by the South Central – Oxford A Research Ethics Committee (18/SC/0448).

Note that full information on the approval of the study protocol must also be provided in the manuscript.

Magnetic resonance imaging

Experimental design

Design type	Event-related design.
Design specifications	In the fMRI study, participants completed 75 trials in the pre-task (outside the MRI scanner) and 216 trials in the main task (inside the MRI scanner). Each trial lasted approximately 16.5 seconds including a variable intertrial interval of 2 to 4 seconds. The offer period was jittered independently of the other events allowing us to examine activity time-locked to effort-based decisions. See Figure 1 for full details of the timing of the different experimental events.
Behavioral performance measures	Choices between work and rest were used as a metric of the willingness to work or rest during the task. Computational modelling was used to summarise choice behaviour and create parametric regressors for fMRI analysis.

Acquisition

Imaging type(s)	Functional and structural.
Field strength	3 Tesla.
Sequence & imaging parameters	A Siemens Prisma 3T MRI scanner was used to acquire multiband T2*-weighted echo planar images (EPIs) with blood oxygen level dependent (BOLD) contrast. The EPI volumes were acquired in an interleaved manner, at an oblique angle (≈30°) to the AC-PC line to reduce signal dropout and the impact of artefacts in frontal regions, and had the following acquisition parameters: voxel size = 2x2x2 mm; slice thickness = 2mm; TE=30 ms; TR = 1570ms; flip angle = 70°; field of view = 216 mm. Subsequent to the functional sequence, a gradient echo field map sequence was used to correct for geometric distortions. Prior to the functional sequence, a structural scan was obtained with voxel size = 1x1x1 mm, slice thickness = 1mm, TE = 3.97ms, TR = 1900ms, flip angle = 8°, field of view = 192mm.
Area of acquisition	Whole brain scan.

Diffusion MRI ☐ Used ☒ Not used

Preprocessing

Preprocessing software	Imaging data was analysed using SPM12 (https://www.fil.ion.ucl.ac.uk/spm). Preprocessing stages included motion correction and correction for spatial distortions by means of bias correction and realignment including the fieldmap, coregistration/segmentation/normalisation, and spatial smoothing using a full-width-half-maximum Gaussian kernel of 8mm.
Normalization	For each participant, the mean of the realigned and unwarped functional images was coregistered to their own structural image. Next, the coregistered structural image was segmented based on standard stereotaxic space (Montreal Neurological Institute, MNI), bias-corrected and normalised to the MNI template. The same normalisation parameters were used to convert the realigned and unwarped functional images into standard space, using non-linear transformations as implemented in SPM.
Normalization template	The MNI template was used as is standard in SPM.
Noise and artifact removal	The six rigid body motion parameters estimated during the realignment step (three translations and three rotations) were added to the design matrices as separate regressors that were not convolved with the HRF to control for nuisance effects resulting from head motion. In addition, functional images were spatially smoothed using an 8mm full-width-half-maximum Gaussian kernel in order to improve the signal-to-noise ratio, and the high-pass filter cut-off during the first-level statistical analyses was set to 128 seconds in order to remove low-frequency noise.
Volume censoring	No volume censoring was used - participants with excessive noise were removed from data analysis.

Statistical modeling & inference

Model type and settings	Univariate models were used. First, we estimated contrasts for each subject and parametric modulator separately with a first-level statistical analysis (fixed effects). We then conducted a second-level statistical analysis to combine data across participants (random effects).
Effect(s) tested	Multiple analyses are performed in the manuscript. The main statistical effects examined were the relation between three parametric regressors defined by three states within our computational model - referred to as recoverable fatigue, unrecoverable fatigue and fatigue-weighted value.

Specify type of analysis: ☐ Whole brain ☐ ROI-based ☒ Both

Anatomical location(s)	The a priori regions of interest (ROIs) were defined anatomically using a mask constructed from the Harvard-Oxford Atlas (for the ventral striatum (nucleus accumbens)) and using a mask defined with respect to the resting-state parecellations of Neubert et al. (2015) (for the bilateral dACC/pre-SMA (areas RCZa, RCZp and pre-SMA). Whole brain anatomical regions were identified by manual inspection with the help of Automated Anatomical Labeling 3 (Rolls et al., 2019; Tzourio-Mazoyer et al., 2002) and Neuromorphometrics, as implemented in SPM12, and Neubert et al. (2015) atlases.

Statistic type for inference (See Eklund et al. 2016)	Voxel-level family-wise error (FWE) correction was used.
Correction	Whole brain family-wise error correction ($p < .05$) and small volume family-wise error correction in predetermined regions of interest ($p < .05$) were used.

Models & analysis

n/a	Involved in the study
☒	☐ Functional and/or effective connectivity
☒	☐ Graph analysis
☒	☐ Multivariate modeling or predictive analysis
